# Complete genome sequence of the hepatitis B virus from hemodialysis patients with occult infection in Bangladesh

**DOI:** 10.1128/mra.01081-25

**Published:** 2025-12-17

**Authors:** Roni Mia, Anandha Mozumder, Parsa Irin Disha, Nashid Sultana Ishi, S. M. Nazmul Hasan, Anupam Das, Chirojit Debnath, Jebin Tasmin, M. Shaminur Rahman, Mohammad Imtiaj Uddin Bhuiyan, Sharmin Akter, S. M. Rashed Ul Islam, Muzahed Uddin Ahmed, Chitta Ranjan Debnath, Md. Golzar Hossain

**Affiliations:** 1 Department of Microbiology and Hygiene, Bangladesh Agricultural University 54492, Mymensingh, Bangladesh; 2 Department of Microbiology, Mymensingh Medical College 469127 , Mymensingh, Bangladesh; 3 Department of Hepatology, Mymensingh Medical College 469127 , Mymensingh, Bangladesh; 4 Department of Microbiology, Jashore University of Science and Technology 421984, Jashore, Bangladesh; 5 Department of Environmental Health Science, University of Georgia 1355, Athens, Georgia, USA; 6 Department of Physiology, Bangladesh Agricultural University, Mymensingh, Bangladesh; 7 Department of Virology, Bangladesh Medical University, Shahbag, Dhaka, Bangladesh; 8 Department of Medicine, Bangladesh Agricultural University, Mymensingh, Bangladesh; Katholieke Universiteit Leuven, Leuven, Belgium

**Keywords:** hepatitis B virus, complete genome, occult infection, hemodialysis, Bangladesh

## Abstract

Hepatitis B virus (HBV) can cause occult infections and has been associated with kidney diseases, particularly in patients undergoing hemodialysis. Here, we report the complete genome sequence of an HBV strain identified in a hemodialysis patient with occult HBV infection in Bangladesh.

## ANNOUNCEMENT

Hepatitis B virus (HBV) infection remains a major global health concern, capable of causing occult hepatitis B infection (OBI). It is also linked to kidney diseases, particularly among patients undergoing hemodialysis (1). HBV belongs to the family *Hepadnaviridae*, genus *Orthohepadnavirus*, and contains a partially double-stranded circular DNA genome of approximately 3.2 kb (2). The virus exhibits substantial genetic diversity and is classified into 10 genotypes (A–J), with further subdivision into subgenotypes (3). Previous report showed a high prevalence of OBI among hemodialysis patients with end-stage renal disease (4). Although HBV infections have been documented in this patient group, and OBI is considered endemic in hospitalized populations, to date, no complete genome sequence of HBV from hemodialysis patients with OBI in Bangladesh has been published (5).

A serum sample was collected from an HBsAg-negative hemodialysis patient with confirmed OBI. Total DNA was extracted using the QIAamp DNA Mini Kit (Qiagen, Germany). The overlapping open reading frames (ORFs) P, S, C, and X were amplified using previously described gene-specific primers (6). Amplified PCR products were purified using a DNA purification kit (GL Sciences, Inc., Tokyo, Japan). The purified products were pooled and subjected to whole-genome sequencing using Illumina technology. Shotgun metagenomic libraries were prepared using a modified iNextEra protocol. DNA tagmentation was performed with Illumina bead-linked transposomes (Illumina DNA Library Prep, Illumina, Inc.), and pooled libraries were sequenced on an Illumina NovaSeq X Plus platform (2 × 150 bp; Novogene, USA). The initial sequencing yielded 22.4 million total reads. Raw paired-end reads were quality-trimmed, and adapters were removed using Trimmomatic (v.0.39), retaining reads >50 bp. Quality control was assessed using FastQC (v.0.11.9). Reads were mapped against the HBV reference genome (GenBank accession no. NC_003977.2; genome length: 3182 bp) using BWA-MEM (v.0.7.17) (7). Resulting alignments were processed using Samtools (v.1.12) (8), FreeBayes (v.1.3.5) (9), and bcftools (v.1.12) (10) to generate a consensus sequence. The sequence provides 100% coverage of the viral genome at a mean depth of 563,787×. The genomes were annotated, and overlapping ORFs were identified by aligning with the reference genome (GenBank accession no. OR769214.1) in Prokka (v.1.14.6) and CLC sequence viewer (v.8). Genotype and subtype were determined using Geno2pheno (https://hbv.geno2pheno.org/) and previously published methods (11). All tools were used with default parameters unless otherwise specified.

The identified full-length circular HBV genome (MGH_HBV-104) was 3,182 bp and contained four overlapping ORFs: P (2,499 bp), S (1,170 bp), C (639 bp), and X (465 bp). The GC content was 48.8%. BLAST analysis showed 99.69% and 99.50% identity to previously reported Bangladeshi HBV strains MGH_HBV-14 (OR769214.1) and BD-HBV08 (MF925364.1), respectively. The strain was classified as genotype D (D2), subtype “ayw.”

This complete genome sequence from hemodialysis patients with occult infection provides important data for understanding HBV genetic diversity in Bangladesh and supports future efforts in antiviral resistance monitoring and molecular epidemiological studies.

## Data Availability

The complete genome sequence has been deposited in GenBank under accession number PV982280. The associated BioSample, BioProject, and SRA accession numbers are SAMN50183278, PRJNA1295711, and SRX29834821, respectively.

## References

[1] Eslami N, Poortahmasebi V, Sadeghi J, Ghotaslou R, Niknafs B, Bannazadeh Baghi H, Ahangar Oskouee M. 2022. Occult hepatitis B infection among hemodialysis in Tabriz, Northwest of Iran: prevalence and mutations within the S region. Can J Infect Dis Med Microbiol 2022:3838857. doi:10.1155/2022/3838857 35800327 PMC9256460

[2] Karayiannis P. 2017. Hepatitis B virus: virology, molecular biology, life cycle and intrahepatic spread. Hepatol Int 11:500–508. doi:10.1007/s12072-017-9829-7 29098564

[3] Hossain MG, Mahmud MM, Rahman MA, Akter S, Nazir K, Saha S, Wada M, Ohsaki E, Honda T, Ueda K. 2020. Complete genome sequence of a precore-defective hepatitis B virus genotype D2 strain isolated in Bangladesh. Microbiol Resour Announc 9:e00083-20. doi:10.1128/MRA.00083-20 32165384 PMC7067952

[4] Shrestha S, Tiwari PS, Pradhan B. 2021. Occult hepatitis B infection in end-stage renal disease patients starting maintenance hemodialysis at a tertiary care hospital: a descriptive cross-sectional study. JNMA J Nepal Med Assoc 59:336–341. doi:10.31729/jnma.5723 34508536 PMC8369594

[5] Chowdhury FR, McNaughton AL, Amin MR, Barai L, Saha MR, Rahman T, Das BC, Hasan MR, Islam KMS, Faiz MA, Al-Mahtab M, Mokaya J, Kronsteiner B, Jeffery K, Andersson MI, de Cesare M, Ansari MA, Dunachie S, Matthews PC. 2021. Endemic HBV among hospital in-patients in Bangladesh, including evidence of occult infection. J Gen Virol 102:001628. doi:10.1099/jgv.0.001628 34328828 PMC8491891

[6] Hossain MG, Islam M, Jeba N, Hasan SMN, Akter M, Mou MJ, Paul SK, Akter S, Khan MK, Saha S, Ahmed MU, Debnath C, Sumon MAU, Salauddin M, Debnath CR. 2024. Complete genome sequence of hepatitis B virus identified from a patient suffering from chronic kidney disease in Bangladesh. Microbiol Resour Announc 13:e0115123. doi:10.1128/mra.01151-23 38624203 PMC11080553

[7] Li H. 2013. Aligning sequence reads, clone sequences and assembly contigs with BWA-MEM. arXiv. doi:10.48550/arXiv.1303.3997

[8] Li H, Handsaker B, Wysoker A, Fennell T, Ruan J, Homer N, Marth G, Abecasis G, Durbin R, 1000 Genome Project Data Processing Subgroup. 2009. The sequence alignment/map format and SAMtools. Bioinformatics 25:2078–2079. doi:10.1093/bioinformatics/btp352 19505943 PMC2723002

[9] Garrison E, Marth G. 2012. Haplotype-based variant detection from short-read sequencing. arXiv. doi:10.48550/arXiv.1207.3907

[10] Danecek P, Bonfield JK, Liddle J, Marshall J, Ohan V, Pollard MO, Whitwham A, Keane T, McCarthy SA, Davies RM, Li H. 2021. Twelve years of SAMtools and BCFtools. Gigascience 10:giab008. doi:10.1093/gigascience/giab008 33590861 PMC7931819

[11] Lusida MI, Nugrahaputra VE, Soetjipto k, Handajani R, Nagano-Fujii M, Sasayama M, Utsumi T, Hotta H. 2008. Novel subgenotypes of hepatitis B virus genotypes C and D in Papua, Indonesia. J Clin Microbiol 46:2160–2166. doi:10.1128/JCM.01681-07 18463220 PMC2446895

